# Supplementary material for: A Non-Coding RNA Network Involved in KSHV Tumorigenesis
Source: Front Oncol. 2021 Jun 16;11:687629. doi: 10.3389/fonc.2021.687629 (PMC8242244; doi:10.3389/fonc.2021.687629)
Supplement: Supplementary file 9 [file DataSheet_1.pdf]

# A non-coding RNA network involved in KSHV tumorigenesis

**Julián Naipauer[1,2&], Martín E. García Solá[2,3,4&], Daria Salyakina[1], Santas Rosario[1], Sion Williams[2,5], Omar Coso[2,3,4], Martín C. Abba[2,6], Enrique A. Mesri[1,2*], Ezequiel Lacunza[2,6*].**

**[&] These authors contributed equally to this work.**

**[*]Co-corresponding authors.**

1 Tumor Biology Program, Sylvester Comprehensive Cancer Center and Miami Center for AIDS Research, Department of Microbiology and Immunology, University of Miami Miller School of Medicine, Miami, Florida, United States of America, 2 UM-CFAR/ Sylvester CCC Argentina Consortium for Research and Training in Virally induced AIDS-Malignancies University of Miami Miller School of Medicine, Miami, Florida, United States of America, 3 Universidad de Buenos Aires, Facultad de Ciencias Exactas y Naturales, Departamento de Fisiología y Biología Molecular, Buenos Aires, Argentina, 4 CONICET-Universidad de Buenos Aires, Instituto de Fisiología, Biología Molecular y Neurociencias (IFIBYNE), Buenos Aires, Argentina, 5 Neurology Basic Science Division, Sylvester Comprehensive Cancer Center; University of Miami Miller School of Medicine, Miami, Florida, United States of America, 6 Centro de Investigaciones Inmunológicas Básicas y Aplicadas, Facultad de Ciencias Médicas, Universidad Nacional de La Plata, La Plata, Argentina.

E-mails:

emesri@med.miami.edu

ez.lacunza@gmail.com

**Supplementary information. (S1 and S2 Figures)**

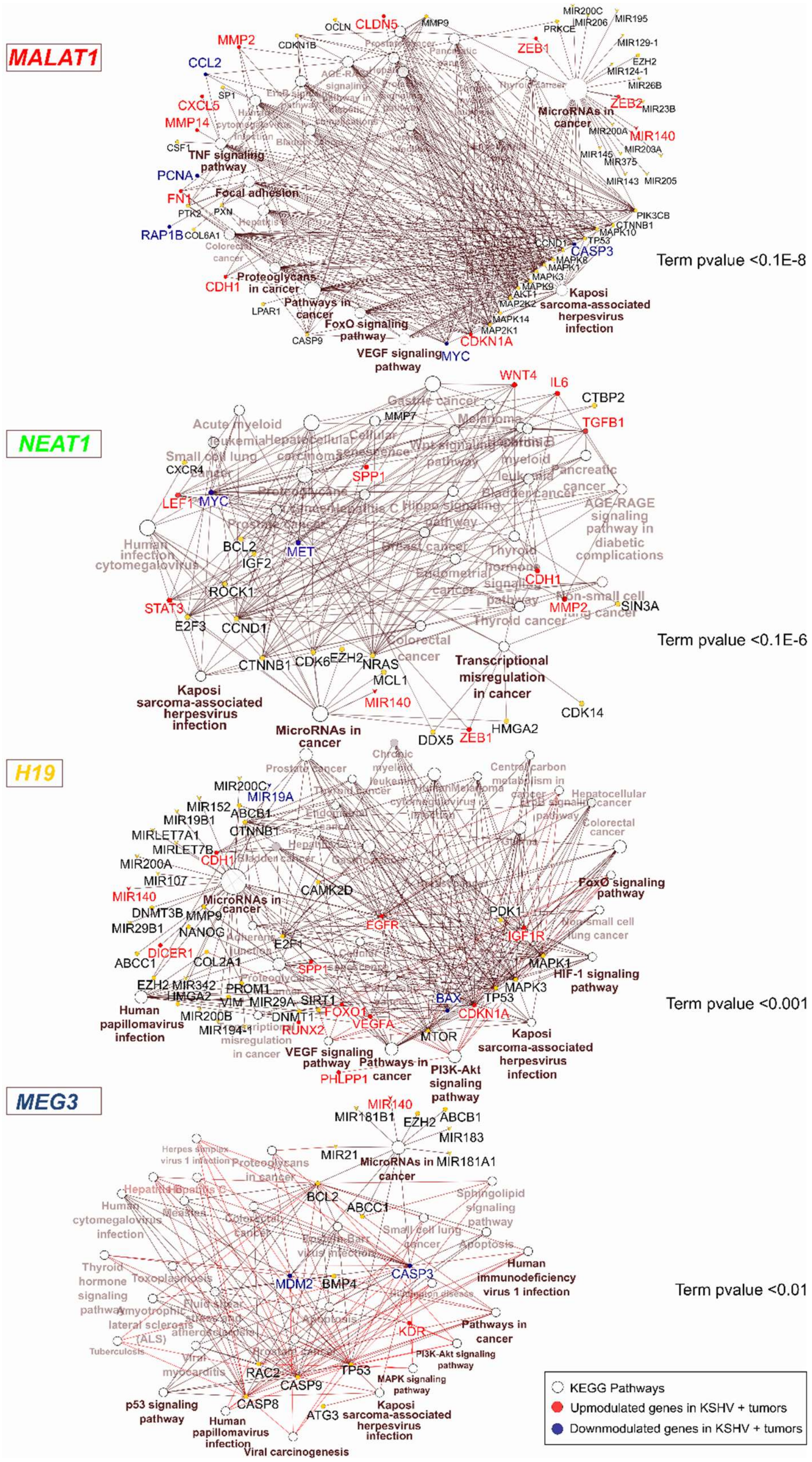


**S1 Figure: LncRNA EVT network and their related pathways.** Highlighted in red and blue are the upregulated and downregulated genes (FC >1.5; pvalue <0.05) in the KSHV+ tumors of the mouse model, respectively.

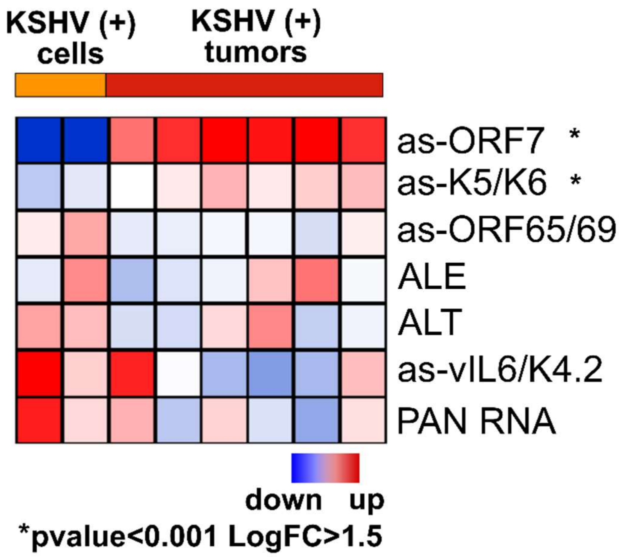


**S2 Figure: Heat map representation for KSHV lncRNAs expression identified in the RNA sequencing data.** DEG analysis showed two lncRNA, as-ORF7 and as-K5/k6, significantly upregulated for the comparison KSHV (+) cells versus KSHV (+) tumors.
